# Development and Characterization of Novel Microsatellite Markers for the Peach Fruit Moth *Carposina sasakii* (Lepidoptera: Carposinidae) Using Next-Generation Sequencing

**DOI:** 10.3390/ijms17030362

**Published:** 2016-03-15

**Authors:** You-Zhu Wang, Li-Jun Cao, Jia-Ying Zhu, Shu-Jun Wei

**Affiliations:** 1 Institute of Plant and Environmental Protection, Beijing Academy of Agriculture and Forestry Sciences, Beijing 100097, China; youzhu0714@163.com (Y.-Z.W.); gmatjhpl@163.com (L.-J.C.); 2 Key Laboratory of Forest Disaster Warning and Control of Yunnan Province, College of Forestry, Southwest Forestry University, Kunming 650224, China; 3 Beijing Key Laboratory for Forest Pest Control, College of Forestry, Beijing Forestry University, Beijing 100083, China

**Keywords:** peach fruit moth, *Carposina sasakii*, simple sequence repeat, next-generation sequencing

## Abstract

The peach fruit moth *Carposina sasakii* is an economically important pest on dozens of fruits from Rosaceae and Rhamnaceae in Northeast Asia. We developed novel microsatellite markers for *C. sasakii* from randomly sequenced regions of the genome using next-generation sequencing. In total, 95,153 microsatellite markers were isolated from 4.70 GB genomic sequences. Thirty-five polymorphic markers were developed by assessing in 63 individuals from two geographical populations. The allele numbers ranged from 2 to 9 with an average value of 4.60 per locus, while the polymorphism information content ranged from 0.075 to 0.696 with an average value of 0.407. Furthermore, the observed and expected heterozygosity varied from 0.000 to 0.677 and 0.062 to 0.771, respectively. The microsatellites developed provide abundant molecular markers for investigating genetic structure, genetic diversity, and existence of host-plant associated biotypes of *C*. *sasakii*.

## 1. Introduction

The peach fruit moth *Carposina sasakii* Matsumura (Lepidoptera: Carposinidae), is an important orchard pest in Northeast Asia [1,2]. In China, this pest was distributed throughout the country, except for Tibet. Its larvae can inflict direct damage on dozens of fruits, including peach, apple, pear, jujube, wild jujube, apricot, hawthorn, and pomegranate [3,4,5,6,7] by boring into fruitage. Differences in number of generations, emergence time of overwintering and diapause generation were found among populations on different host species [8,9], likely leading to low gene flow among host-plant populations. Thus, several studies attempted to reveal the differentiation of those moths occurring on different host plants [10,11].

To date, three types of molecular marker have been used to examine the existence of host biotypes in *C. sasakii*. Using esterase isozyme, Hua *et al.* (1995) [11] reported that there is nearly no differentiation in isozyme-spectra between *C. sasakii* collected from jujube and wild jujube; however, populations collected from above two hosts were obviously different from those collected from apple orchard. Using RAPD (random amplified polymorphic DNA) to compare the populations collected from six kinds of host plants, including apple, hawthorn, peach, apricot, jujube, and wild jujube, Xu and Hua revealed that there were remarkable genetic differentiation between populations from apricot and those from other hosts [12]. Although a recent study using one region of mitochondrial DNA (mtDNA) sequences of cytochrome coxidase subunit I (COI) found that there was no evidence for associations between the variation of populations and host plants, the genetic differentiation showed significant correlation with the geographical distance [13]. Varied genetic markers used in the studies obviously lead to different results. To address this issue, more polymorphic and stable molecular markers are required.

Microsatellite is a kind of special sequence comprised by tandem repeats of one to six nucleotides. It always has high polymorphism, widely dispersed in both coding and noncoding regions of all prokaryotic and eukaryotic genomes [14]. Due to their codominant inheritance, high polymorphism, easy detection by polymerase chain reaction (PCR), and broad distribution in the genome, microsatellites are widely used for population genetic studies [15,16,17].

The traditional approach of microsatellite development, such as an enriched library followed by gene cloning, is time-consuming and labor-intensive. New approaches based on next-generation sequencing can be a good alternative. With the advantage of this technology, it is possible to develop a huge number of microsatellites, which are capable of generating tens of millions of short DNA sequence reads at a relatively low cost [18,19,20,21].

In the present study, we aimed to isolate microsatellites for *C*. *sasakii* from randomly obtained genomic sequences. This is the first report of novel microsatellites for *C*. *sasakii*. The markers developed will be helpful in investigating genetic structure, genetic diversity, and existence of host-plant associated biotypes of *C*. *sasakii*.

## 2. Results and Discussion

### 2.1. Microsatellite Marker Development

We generated 4.70 GB paired-end (PE) sequences with read length of 300 base pairs (bp), including 15,725,132 reads from a 500 bp insert DNA library constructed by Illumina MiSeq system. Raw data sequences were submitted to the National Center for Biotechnology Information (NCBI) Short Read Archive under accession number SRP068817. After removing low quality reads using SolexaQA software [22], the remaining high-quality reads were assembled into 1,902,994 contigs by SOAPdenovo2 [23]. They were with mean size of 252 bp and N50 of 286 bp, which are much shorter compared to a similar study in *Dorcus hopei* (Coleoptera) (N50 = 1218) [24]. This might be due to the method of assembly and the coverage of sequencing reads. However, the number of primer pairs designed in our study is reasonable (totally 8074 primer pairs / 479 Mb), as in other studies [19,24,25,26].

A total of 95,153 microsatellite loci were discovered using MSDB version 2.4.3 software (http://msdb.biosv.com/) from the assembled contigs, which will be provided upon request. The detected microsatellites included 54,559 (57.34%) dinucleotide, 34,957 (36.74%) trinucleotide, 5591 (5.88%) tetranucleotide, and 46 (0.05%) pentanucleotide repeats (Table 1). There are no hexanucleotide repeats found under our searching conditions of microsatellite loci (a minimum of 25, 5, 5, 5, 5 and 5 repeats were used to identify the mono-, di-, tri-, tetra-, penta-, and hexanucleotide motifs, respectively). Dinucleotide repeats are more than the higher order motif, which is in agreement with the previous report of Arthropoda in insects like *Aphis glycines* (Hemiptera) [27] and *Coccinella septempunctata* (Coleoptera) [28], and in species of Arachnida [29]. According to the distribution of microsatellite (Table 1), it seems that the quantity of loci decreases followed with the increase of corresponding motif repeats.

Sixty-four primer pairs designed according to the sequences that are flanking trinucleotide repeats were selected for initial validation in eight individuals. Of them, 35 loci have polymorphic amplifications, 16 loci were monomorphic and 13 primer pairs did not produce any visible amplicon. These polymorphic loci can serve as candidate markers for future research, such as genetic diversity and relatedness analysis of different populations.

### 2.2. Characteristics of Validated Microsatellite Loci

**Figure 1:**
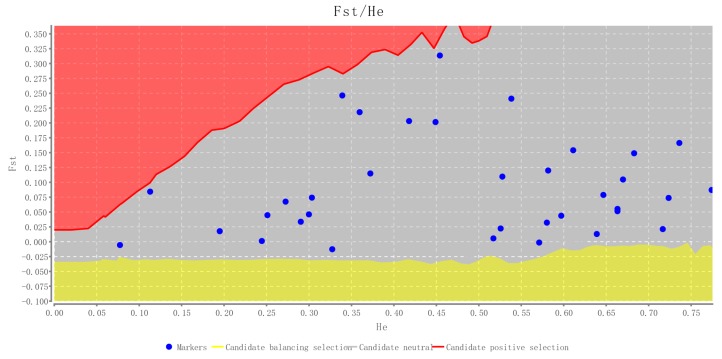
Potential candidates for selection. Loci located in the red region are candidates for positive selection, grey region for neutral, and yellow region for balancing selection. All of the 35 loci are under neutral expectations.

The polymorphic loci obtained were assessed with two *C*. *sasakii* natural populations, including 31 individuals from Beijing and 32 individuals from Hubei province, China (Table 2 and Table S1). The 35 microsatellite markers had allele numbers ranging from 2 to 9 with an average value of 4.60 per locus. The polymorphism information content (PIC) revealed a range from 0.075 to 0.696 with an average value of 0.407. The observed (*H_O_*) and expected (*H_E_*) heterozygosity ranged from 0.000 to 0.677 and 0.062 to 0.771, respectively. The inbreeding coefficient (*F_IS_*) ranged from −0.240 to 1.00. The significantly high *F_IS_* in locus CS21, CS38 and CS82 might be caused by the low *H_O_*, rather than sampling bias since most loci showed low *F_IS_* in the two populations. The loci CS31 and CS33 showed significant linkage disequilibrium only across Beijing population (corrected by Holm’s correction, *p* < 0.05). It is speculated that the linkage disequilibrium observed at certain loci in some populations may be due to substructure of population or bottleneck [30]. Eight loci in Beijing population and 13 loci in Hubei population significantly deviated from Hardy-Weinberg equilibrium (HWE), while 5 loci (CS05, CS17, CS21, CS29 and CS82) showed significant value in the both tested populations. The loci deviated from HWE might be resulted by heterozygote deficiency, because *H_O_* is much lower than *H_E_* in these loci (Table 2). Heterozygote deficiency can be caused by the Wahlund Effect [31] or the presence of null alleles, for which Lepidoptera species are notorious [32,33,34,35,36]. It was considered that the present of null alleles is very common in this order due to the flanking region with repetitive sequences and multiple copies of loci [37,38,39]. Random sequences of *C*. *sasakii* genome obtained by the Illumina MiSeq system may cover coding regions. Thus, they are probably linked to sites under selection, which cannot reflect facticity of population diversity and structure. A neutrality test was done with all of the 35 loci. Interestingly, all of the loci were under neutral expectations (Figure 1). Therefore, deviating from HWE is not necessarily due to the characteristics of loci. It may imply the distinct population structure, biological property of the species, or just sampling error, e.g., examined individuals from the same egg brood can also lead to deviation from HWE [40].

**Figure 2:**
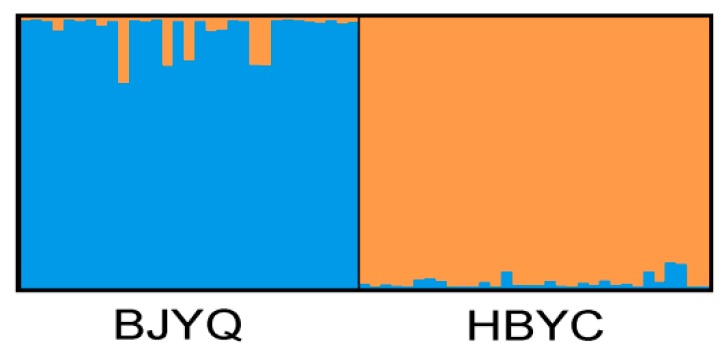
Population structure of *K* = 2 inferred by Bayesian clustering approaches based on 35 microsatellite markers. BJYQ: population of Yanqing from Beijing; HBYC: population of Yichang from Hubei province.

The population structure of *C*. *sasakii* was inferred with the dataset of 35 microsatellite markers. The 63 individuals from two geographic populations were divided into two clusters. As can be clearly seen in Figure 2, there are genetic differences between two populations, indicating that the microsatellite markers validated could be used to discriminate geographic populations and other genetic study of *C*. *sasakii*. 

## 3. Materials and Methods

### 3.1. Sample Collection and DNA Extraction

A total of 63 larvae were collected from two geographic regions in China, of which 31 samples were from Yanqing of the Beijing (N 40°27′20.05″, E 115°58′8.14″), named BJYQ, and 32 specimens came from Yichang of Hubei province (N 30°41′39.43″, E 111°16′50.77″), named HBYC. Additionally, eight individuals from eight sampled sites were used for the initial test. Samples were stored in ethanol absolute and frozen at −80 °C prior to use. Genomic DNA were extracted from half of an individual larva using DNeasy Blood & Tissue Kit (QIAGEN, Hilden, Germany), according to the manufacturer’s instructions.

### 3.2. Sequencing, Microsatellites Searching and Primer Design

One larva of *C*. *sasakii* from Beijing was used to prepare the library with the Illumina TruSeq DNA PCR-Free HT Library Prep Kit (Illumina, San Diego, CA, USA), and then sequenced on a Illumina MiSeq Sequencer using the MiSeq Reagent Kit v3 (Illumina, San Diego, CA, USA). Generated genomic sequences were assembled by SOAPdenovo program [23].

The microsatellite isolation from the genomic sequences and primer design for loci was conducted in the software QDD [41]. The searching criteria were as follows: at least six motif repeats for target microsatellites, and PCR product lengths ranged between 90 and 350 bp. For primer design, the annealing temperature ranged from 52 to 68 °C, and the difference in annealing temperature in one pairwise primer was <5 °C. The remaining parameters were at default settings.

### 3.3. Primer Testing and Polymorphism Detection

Firstly, in order to improve efficiency and lower cost, we added a PC tail (Primer tail C) (5′ CAGGACCAGGCTACCGTG 3′) to the 5′ end of the candidate forward primer [42]. Eight larvae of *C*. *sasakii* from eight different populations were used for the initial test. Amplification was carried out in a final volume of 10 μL, containing 0.5 μL (12.5 ng) of template DNA, 5 μL of Master Mix (Promega, Madison, WI, USA), 0.25 μL of forward primer (modified by the PC tail) at a final concentration 0.25 µM, 0.25 μL (10 µM) of reverse primer at a final concentration 0.25 µM, and 4 μL of ddH_2_O. The amplification program was as follows: 4 min at 94 °C; 35 cycles of 30 s at 94 °C, 30 s at 56 °C, and 45 s at 72 °C, with a final 10-min extension at 72 °C. PCR products were visualized on agarose gel (1.5%) electrophoresis. This step was taken to screen primers that can amplify PCR fragment.

Secondly, primers selected in previous steps were tested using a capillary sequencer. Amplification was performed in a final volume of 10 μL, containing 0.5 μL (12.5 ng) of template DNA, 5 μL of Master Mix (Promega, Madison, WI, USA), 0.08 μL of forward primer (modified by the PC tail) at a final concentration 0.08 µM, 0.16 μL of reverse primer at a final concentration 0.16 µM, 0.32 μL of PC tails modified by fluorescence (FAM (blue), HEX (green), and ROX (red)) including different color at a final concentration 0.32 µM, and 3.94 μL of ddH_2_O. The amplification program was the same as above. The ABI 3730xl DNA Analyzer (Applied Biosystems, Foster, CA, USA) was used to analyze the amplified PCR fragments with the GeneScan 500 LIZ size standard (Applied Biosystems).

Finally, marker primers screened out by the first two steps were validated in 63 samples from two regions. Amplification mixture, amplification program, and analysis of PCR fragments were the same as the second step.

### 3.4. Statistical Analysis

Genotyping data was identified, and errors were corrected by MICRO-CHECKER [43]. Diversity statistics including allele frequencies, *Ho*, *He* and PIC were estimated by the macros Microsatellite Tools [44]. Tests for linkage disequilibrium among loci within each population and deviation from HWEat each locus/population pair, and estimation of *F_IS_* for each population, were performed in GENEPOP v4.0 (Applied Biosystems). Additionally, the null allele test was conducted with FREENA [34]. The program LOSTAN [45] was used to detect putative loci potentially under selection with two options: neutral mean *F_ST_’* and force mean *F_ST_’*. Corresponding sequences of polymorphic loci were screened using BLASTx and BLASTn in the NCBI database (http://www.ncbi.nlm.nih.gov/). Population differentiation was investigated using the Bayesian clustering approach implemented in the program STRUCTURE, version 2.3.3 [46]. Simulations were run for 200,000 Markov chain Monte Carlo with a burn-in of 100,000 iterations under admixture ancestry and correlated allele frequency models. We performed 15 independent runs for each *K* (from 1 to 6) to confirm consistency across runs. The most accurate number of groups (*K*) was visually examined when plotting *K* against delta-*K* and using the Evanno method in the online program STRUCTURE HARVESTER [47].

## 4. Conclusions

We characterized and developed microsatellite markers for *C. sasakii* from random regions of the genome generated by using next-generation sequencing. The loci assessed in our study could reveal the genetic structure in two geographical populations. This method provides fast way for high throughput development of microsatellite markers from non-model species without reference genome.

## Figures and Tables

**Table 1:** Distribution of microsatellites with different motifs and repeat numbers in *Carposina sasakii.*

Repeat Motif	Number of Repeats								Total Frequency (%)
	5	6	7	8	9	10	11	12	
AC/GT	7521	2765	1747	1543	1433	720	76	2	16.612
AG/CT	2080	862	512	387	415	340	72	2	4.908
AT/AT	14,053	5758	4165	4491	3380	732	42	2	34.285
CG/CG	1325	109	12	4	3	3	1	2	1.533
AAC/GTT	92	34	18	3					0.154
AAG/CTT	2074	747	142	3					3.117
AAT/ATT	8333	4004	438	3					13.429
ACC/GGT	45	12	6	1					0.067
ACG/CGT	457	121	38	3					0.651
ACT/AGT	218	71	50	3					0.359
AGC/CTG	77	27	14	3					0.127
AGG/CCT	49	7	7	1					0.067
ATC/ATG	11,707	5017	435	3					18.036
CCG/CGG	436	162	93	3					0.729
AAAC/GTTT	257	4							0.274
AAAG/CTTT	355	4							0.377
AAAT/ATTT	1798	4							1.894
AACC/GGTT	14	4							0.019
AACG/	1	1							0.002
AACT/AGTT	42	4							0.048
AAGG/CTTC	8	2							0.011
AAGT/ACTT	62	4							0.069
AATC/GATT	146	4							0.158
AATG/CATT	283	4							0.302
AATT/AATT	68	3							0.075
ACAG/CTGT	112	4							0.122
ACAT/ATGT	1448	4							1.526
ACCT/AGGT	114	3							0.123
ACGC/ACGC	20	4							0.025
ACGG/CGTC	51	4							0.058
ACGT/ACGT	3								0.003
ACTC/GTGA	20	3							0.024
AGAT/ATCT	473	4							0.501
ATCC/ATGG	216	4							0.231
ATGC/ATGC	6	1							0.007
AGGC/CCTG	10	2							0.013
CGAG/CTCG	2								0.002
CGGC/CGGC	3								0.003
CTAG/CTAG	3								0.003
GACT/TCAG	2	1							0.003
GCAA/GCAA	2								0.002
OTHERS	46								0.048
DNR	24,979	9494	6436	6425	5231	1795	191	8	57.338
TNR	23,488	10,202	1241	26					36.738
TTNR	5519	72							5.876
PNR	46								0.048

DNR: dinucleotide repeats; TNR: trinucleotide repeats; TTNR: tetranucleotide repeats; PNR: pentanucleotide repeats.

**Table 2:** Characteristics of 35 microsatellite loci validated in 63 individuals of *Carposina sasakii.*

Locus	Dye	Repeat Motif	Primer Sequence (5′–3′)	Allele No.	Size Range (bp)	*HWE*		*r*		*H_O_*		*H_E_*		*F_IS_*		PIC
						Beijing	Hubei	Beijing	Hubei	Beijing	Hubei	Beijing	Hubei	Beijing	Hubei	
CS03	ROX	(AGT)6	F: TAAAAGCGATTCGTTGGGAC	5	209–218	0.608	1.000	0.000	0.000	0.419	0.125	0.387	0.122	−0.086	−0.029	0.250
			R: ATGGCGTCATATCTTCGACC													
CS04	FAM	(ACT)6	F: TTCCGTGCATGTCGTAAGAG	6	120–139	0.012	0.016	0.100	0.011	0.484	0.406	0.655	0.468	0.265	0.133	0.531
			R: CGCGTTTAGCATCAATCTCA													
CS05	HEX	(ACG)6	F: ACACTAGTTGAGTGATTTCAACCG	5	101–113	0.000	0.000	0.323	0.269	0.097	0.188	0.622	0.631	0.847	0.706	0.572
			R: GCATCTGGCTAGATTCTGATGA													
CS06	HEX	(CCG)6	F: ACCGACCAGTCCATTCGAT	4	106–123	0.460	0.856	0.000	0.005	0.613	0.469	0.539	0.489	−0.139	0.041	0.412
			R: CTCCTTAGGTCTCTGCGTCG													
CS07	HEX	(AAT)6	F: AGCAGCCTGCATCCAACC	9	99–122	0.738	0.000	0.000	0.106	0.581	0.581	0.643	0.771	0.098	0.250	0.696
			R: ACACACTCCCAATTCGCTTC													
CS101	HEX	(AAC)6	F: TTGGTTCATGGATCTAGGAGG	4	104–115	0.007	0.006	0.145	0.132	0.161	0.219	0.309	0.354	0.483	0.385	0.304
			R: TCCTAAGTCTACCTAACTTTATGTGTT													
CS102	FAM	(AGT)6	F: CCGTAATAATTCGACACAAGCA	5	131–147	1.000	0.004	0.000	0.159	0.226	0.219	0.211	0.448	−0.071	0.516	0.325
			R: CCTATACTCGTATACTTAAACAACTGA													
CS103	HEX	(AAC)6	F: AGTATCAAAAGAAACCCCTAA	4	111–120	1.000	0.700	0.011	0.036	0.355	0.594	0.373	0.661	0.049	0.104	0.506
			R: ATCGGCATTATTTGTAAGGT													
CS11	HEX	(AAG)6	F: CCTCGTATTAGATTAGGCGGAA	4	95–112	1.000	0.000	0.000	0.200	0.065	0.250	0.063	0.560	−0.017	0.558	0.343
			R: CCCAAGTTGAATGGGAACAG													
CS14	HEX	(AGT)6	F: TGCGACAAAATGCCAGAATA	6	106–136	0.020	0.952	0.129	0.000	0.355	0.594	0.590	0.554	0.403	−0.074	0.489
			R: GCCGATGTATTCTAATGAAGCC													
CS17	HEX	(AAG)6	F: CTCAAGAGTTCTATATACGGGG	5	102–117	0.000	0.001	0.294	0.170	0.233	0.219	0.751	0.448	0.693	0.516	0.592
			R: GGCGATGGGATAGCTGTTAC													
CS18	HEX	(AAT)6	F: AGATAGCTCGTTGACAAAGTT	3	111–117	0.402	0.000	0.041	0.183	0.194	0.125	0.228	0.344	0.155	0.640	0.272
			R: TGTTTTGGAAGCAACAAACG													
CS19	HEX	(AGT)6	F: CCAATGTGTCGTACAACGTG	7	113–134	0.291	0.015	0.062	0.089	0.516	0.438	0.631	0.561	0.184	0.222	0.568
			R: CCTCAAGTAAATATAATCAGGGCG													
CS20	FAM	(ACT)6	F: CAAATCCTTGGCAATGTGAA	4	109–126	0.030	0.000	0.076	0.224	0.462	0.156	0.646	0.496	0.290	0.688	0.478
			R: AGAAAAGATTCACCTGCGCT													
CS21	FAM	(ACT)6	F: CGCATTTGCTACTCACCTGT	4	105–120	0.000	0.000	0.201	0.248	0.000	0.063	0.178	0.383	1.000	0.839	0.256
			R: ACTTACATTCACGTTGCCCA													
CS22	FAM	(CCG)6	F: GTAACGAGCGCAATTGATGA	3	122–128	0.050	1.000	0.108	0.000	0.032	0.063	0.094	0.062	0.659	−0.008	0.075
			R: CGCGCTAATCTGGTTAATACG													
CS24	ROX	(CCG)6	F: TCTAAGGAGTGTCCGAAGGC	2	247–248	1.000	1.000	0.000	0.013	0.452	0.469	0.444	0.496	−0.017	0.055	0.373
			R: TCAAGTACCGTGTGCGGATA													
CS26	FAM	(CCG)6	F: ACCCGAGTAAAGACCCGACT	4	123–135	0.000	0.105	0.272	0.097	0.129	0.065	0.535	0.182	0.762	0.649	0.360
			R: TGTTAACCCTAGAAGGCCCG													
CS28	FAM	(ACT)6	F: GCTGGTGTGGATGGCATAGT	7	126–147	0.023	0.061	0.082	0.099	0.484	0.438	0.637	0.591	0.243	0.263	0.615
			R: AACTTCGAATTTCCATTGCG													
CS29	FAM	(ACC)6	F: TCGGTCACGTTATTTTAGCAA	9	89–147	0.000	0.000	0.173	0.266	0.290	0.290	0.504	0.525	0.428	0.451	0.494
			R: CATGGTCAGTGCTAGGCAGA													
CS31	FAM	(ACT)6	F: CGGACTTCTGAAACCGTGAT	6	129–148	0.086	0.000	0.028	0.137	0.484	0.484	0.563	0.698	0.143	0.310	0.601
			R: GCCAATTCAGTTATGAGGGC													
CS32	FAM	(AGG)6	F: CTAGGTACACCAATCGGCCA	2	134–137	0.054	0.495	0.111	0.037	0.194	0.438	0.317	0.500	0.394	0.127	0.360
			R: GCTGCCATTTCACCAGTCTT													
CS33	FAM	(ACT)6	F: AATAGGGCTCCTCCACACCT	8	130–156	0.392	0.706	0.030	0.003	0.677	0.531	0.769	0.571	0.121	0.071	0.643
			R: GATCTGCAAATCTGCCTGTG													
CS34	FAM	(AGT)6	F: CGCCCTAGACGAACCTACAC	4	130–143	0.587	1.000	0.026	0.000	0.258	0.219	0.283	0.205	0.091	−0.069	0.227
			R: GCCTATGTTCAGCAGAAGACG													
CS35	ROX	(AAG)6	F: CAAAGATAATGTACAAAGACGTG	5	113–142	0.001	0.040	0.215	0.121	0.269	0.531	0.652	0.750	0.592	0.296	0.655
			R: CAACTGTCTGCAACACAGCA													
CS36	ROX	(CCG)6	F: CACCGATTTGTTTTATCGCA	7	138–159	0.284	1.000	0.025	0.000	0.581	0.063	0.604	0.062	0.039	−0.008	0.351
			R: GGCGCTAATGTCTACCCTCA													
CS37	ROX	(ACC)6	F: TAAGAAGATCCTCGCCCAGA	2	145–148	0.159	0.300	0.081	0.000	0.097	0.406	0.151	0.329	0.362	−0.240	0.215
			R: TACATCGTTGTAGGACCGCC													
CS38	ROX	(AGC)6	F: CAAACAAATTATCCGCGTCC	3	147–153	0.022	0.001	0.140	0.176	0.000	0.000	0.148	0.235	1.000	1.000	0.181
			R: GACAGAAACAATAACAACGACGA													
CS41	ROX	(AAC)6	F: CCACTGGGCTATCACTGCTAT	6	140–168	0.118	0.132	0.040	0.052	0.581	0.281	0.664	0.360	0.128	0.221	0.509
			R: TGCAACAGTGACATCACAAGA													
CS44	ROX	(AGT)6	F: AGTGGGCGCCACCTGCAT	3	149–155	1.000	NA	0.000	0.001	0.226	0.000	0.207	0.000	−0.094	NA	0.102
			R: CCATCTTTGGCTCAGAAAGC													
CS45	ROX	(ACT)6	F: TGGCCGTTATATCATCCACA	2	155–158	1.000	1.000	0.000	0.000	0.065	0.469	0.063	0.448	−0.017	−0.047	0.254
			R: GGTAGTCCTGGTCAGAGGCA													
CS47	ROX	(AGT)7	F: ACCGGTATTGCTGTATTTGT	5	151–163	0.001	0.764	0.163	0.000	0.400	0.625	0.666	0.592	0.404	−0.056	0.573
			R: CAATTTGTGATTAGGTATTTGTTTCAA													
CS48	ROX	(AAT)6	F: TGTAGCAGTCAAGGTCACGG	3	156–162	0.048	0.000	0.071	0.219	0.484	0.194	0.666	0.497	0.277	0.614	0.556
			R: CGCTATAAAAGTGAACGGCG													
CS53	ROX	(AAG)6	F: TCACGTAACCGTCTGGTTCA	3	137–176	1.000	0.802	0.000	0.015	0.097	0.438	0.094	0.469	−0.035	0.068	0.274
			R: TCGTCTTTTCTTTCCATCGG													
CS82	HEX	(AGT)6	F: AAAGGCAGATTAACCGACTAGTGT	2	89–106	0.000	0.000	0.293	0.198	0.000	0.000	0.389	0.173	1.000	1.000	0.247
			R: AAATATTTTCGCGTTCATTTCG													

F: forward primer; R: reverse primer; r: frequency of null allele; *H_O_*: observed heterozygosity; *H_E_*: expected heterozygosity; PIC: polymorphism information content; *F_IS_*: inbreeding coefficient; *HWE*: exact *p*-value of Hardy-Weinberg Equilibrium; BLASTx/BLASTn: results of BLASTx/BLASTn. NA: not available.

## Supplementary Materials

The following are available online at www.mdpi.com/1422-0067/17/3/362/s1.
