# Supplementary material for: Development and Characterization of Novel Microsatellite Markers for the Peach Fruit Moth Carposina sasakii (Lepidoptera: Carposinidae) Using Next-Generation Sequencing
Source: Int J Mol Sci. 2016 Mar 15;17(3):362. doi: 10.3390/ijms17030362 (PMC4813223; doi:10.3390/ijms17030362)
Supplement: Supplementary file 1 [file ijms-17-00362-s001.pdf]

Supplementary Materials: Development and Characterization of Novel Microsatellite Markers for the Peach Fruit Moth *Carposina sasakii* (Lepidoptera: Carposinidae) Using Next-Generation Sequencing

You-Zhu Wang, Li-Jun Cao, Jia-Ying Zhu and Shu-Jun Wei

Table S1. Annotated results for the 35 examined microsatellite loci using BLASTx and BLASTn against NCBI database.

Locus	Gene Annotation; GenBank Accession Number	E Value
CS03	Alpha2 macroglobulin isoform2 (*Operophtera brumata*); KOB65313.1	6.00×10^{-4}
CS07	PREDICTED: serine/threonine-protein kinase par-1-like isoform X3 (*Bombyx mori)*; XP 012549778.1	2.00×10^{-3}
CS14	hypothetical protein (*Ralstonia pickettii*); WP 022534306.1	3.20
CS19	hypothetical protein (*Ralstonia pickettii*); WP 022534306.1	3.20
CS24	RNA-binding protein lark (*Danaus plexippus*); EHJ73625.1; / Bombyx mori mRNA,clone: fmgV18J03; AK378213.1	2.00×10^{-142}
CS29	PREDICTED: uncharacterized protein LOC105396690 (*Plutella xylostella*); XP_011566999.1	3.00×10^{-7}
CS32	PREDICTED: uncharacterized protein LOC105841869 (*Bombyx mori*); XP_012547039.1	6.50×10^{-2}
CS37	PREDICTED: uncharacterized protein LOC106130656 (*Amyelois transitella*); XP_013185007.1	6.00×10^{-3}
CS44	PREDICTED: bone sialoprotein-binding protein (*Amyelois transitella*); XP_013193275.1	7.00×10^{-1}

Note: there is not BLAST result for the remaining loci of CS04, CS05, CS06, CS101, CS102, CS103, CS11, CS17, CS18, CS20, CS21, CS22, CS26, CS28, CS31, CS33, CS34, CS35, CS36, CS38, CS41, CS45, CS47, CS48, CS53, CS82.
